# Machine learning-based prediction of hemodynamically significant patent ductus arteriosus in preterm neonates: a pioneering insight

**DOI:** 10.3389/fped.2025.1727418

**Published:** 2025-12-02

**Authors:** Oguzhan Ay, Sezgin Gunes

**Affiliations:** 1 Department of Pediatrics, Division of Pediatric Cardiology, Izmir Democracy University Buca Seyfi Demirsoy Teaching and Research Hospital, Izmir, Türkiye; 2 Department of Pediatrics, Division of Neonatology, Izmir City Hospital, Izmir, Türkiye

**Keywords:** patent ductus arteriosus, premature infants, Random Forest model, machine learning, gestational week, birth weight, F1 score

## Abstract

**Background:**

Hemodynamically significant patent ductus arteriosus (hPDA) in premature infants is a common congenital cardiac anomaly associated with substantial morbidity and mortality. Traditional diagnostic methods like echocardiography face challenges such as expertise requirement and inconsistent accessibility. This study investigates the efficacy of the Random Forest machine learning model in predicting hPDA in premature infants, aiming to provide a non-invasive, objective, and reliable alternative.

**Methods:**

This retrospective study analyzed data from 657 premature infants hospitalized between 2014 and 2019. Patients were categorized into hPDA and asymptomatic PDA (aPDA) groups. The Random Forest classification model, implemented in JASP software, utilized prenatal, natal, and postnatal clinical data, including gestational week, birth weight, and the need for resuscitation at birth. Model performance was assessed using metrics such as accuracy, Area Under the Curve, F1 score, Matthews Correlation Coefficient, recall, precision, and feature importance.

**Results:**

The Random Forest model demonstrated strong predictive performance, achieving a test accuracy of 91.7%, an AUC of 0.950, an F1 score of 0.923, and an MCC of 0.775. Notably, the recall for the hPDA group was 100%. Gestational week, birth weight, and the need for resuscitation at birth were identified as the most significant predictors. The model also revealed complex relationships, showing variables deemed statistically insignificant by classical methods (e.g., gender, 5th-minute APGAR score, oligohydramnios) to be significant within the Random Forest framework.

**Conclusions:**

The Random Forest model effectively predicts hPDA risk in premature infants, offering superior predictive power compared to classical statistical analyses. This approach has the potential to enhance early detection, facilitate timely interventions, and support personalized treatment strategies, thereby improving patient outcomes. Further validation through large-scale, multi-center prospective studies is essential for its integration into clinical practice.

## 1 Introduction

Patent ductus arteriosus(PDA) in premature infants constitutes a prevalent congenital cardiac anomaly linked to substantial morbidity and mortality (1). More precisely, hemodynamically significant PDA commonly affects premature neonates during the initial days of life and has the potential to induce severe systemic consequences, particularly in very low birth weight and highly premature infants (2, 3). As a result, this condition is associated with a range of comorbidities, including chronic lung disease, bronchopulmonary dysplasia, necrotizing enterocolitis, impaired renal function, intraventricular hemorrhage, and elevated mortality rates (4). Consequently, the timely and precise detection of patent ductus arteriosus in premature infants is paramount for mitigating potential complications and enhancing clinical management strategies (5).

Among the traditional diagnostic methods used for premature PDA, echocardiography is considered the gold standard (6). However, this method also presents certain diagnostic challenges and limitations (5). The most significant of these challenges include the requirement for high expertise and its inconsistent accessibility (1). These limitations particularly hinder the continuous monitoring of PDA in premature infants within intensive care units, where hemodynamic status is dynamically changing (3). Consequently, there is a growing need for non-invasive, objective, and reliable alternative methods that can support clinical decision-making processes and enhance diagnostic accuracy (7). In this context, machine learning algorithms offer the potential to analyze extensive clinical data, uncover complex relationships, and develop predictive models (8).

Despite the availability of diverse therapeutic modalities, including pharmacological interventions and surgical procedures and transcatheter closure the ongoing debate and lack of a definitive consensus on the most appropriate management approach for premature PDA continue to persist in current neonatological practice (3, 9–11). This absence of a standardized strategy contributes to divergent treatment paradigms and variable clinical outcomes, further exacerbated by insufficient investigation into the utility of echocardiographic variables for predicting clinical outcomes, especially in the early stages (3).

There is no international consensus on the diagnosis and management of hemodynamically significant patent ductus arteriosus (12–14). This situation primarily stems from the insufficient investigation into the utility of echocardiographic variables for predicting clinical outcomes, especially in the early stages (4, 15). This ambiguity leads to divergent treatment approaches and outcomes, underscoring the necessity for a standardized approach to PDA in premature infants (3, 16). In this context, machine learning algorithms, particularly the Random Forest model, may offer a potential solution for the early prediction of hemodynamically significant PDA, independently of PDA treatments.

This study rigorously investigates the efficacy of the Random Forest model for accurately predicting hemodynamically significant PDA in premature infants. By leveraging a comprehensive dataset comprising pre-delivery, peri-delivery, and post-delivery clinical data alongside physical examination findings from neonates monitored in the neonatal intensive care unit, this research seeks to overcome current diagnostic limitations and facilitate earlier, more targeted interventions.

## 2 Materials and methods

### 2.1 Data collection

This study was constructed from retrospective data with the objective of predicting hemodynamically significant patent ductus arteriosus in premature infants. The model encompasses historical data from the prenatal, natal, and postnatal periods, alongside clinical metrics and physical examination results. Data for this study were sourced from 657 patients, hospitalized with a diagnosis of prematurity in the Neonatal Intensive Care Unit of İzmir Medical Point hospital from 2014 to 2019. Patients in the neonatal intensive care unit were monitored by a neonatology specialist, and the diagnosis of PDA was confirmed by a pediatric cardiology specialist. Adhering to Turkish Neonatology Guidelines, the approach to hemodynamically significant PDA (hPDA) in these patients was established through a consensus involving neonatology and pediatric cardiology specialists (5). According to the guideline, treatment for hPDA was initiated in patients presenting with specific clinical and echocardiographic findings. Clinically, these findings included systemic hypotension, pulmonary hyperperfusion, systemic hypoperfusion, dependence on respiratory support, and the development of metabolic acidosis. Echocardiographically, the criteria comprised an LA/Ao ratio >1.5, a PDA diameter >2 mm, abnormal diastolic flow patterns, retrograde flow, ductal steal, and left atrial enlargement (5). The detailed data acquisition process involved gathering each patient's demographic profile, prenatal narrative, birth records, postnatal clinical trajectory, and laboratory analyses.

### 2.2 Patients groups

PDA was present in all patients' initial echocardiograms. Patients were divided into two groups according to their follow-ups based on echocardiographic and clinical findings. The first group consisted of premature infants diagnosed with hPDA, while the second group included patients in whom hemodynamic PDA was not detected by echocardiography or clinical findings, cases of spontaneous closure during hospitalization, or those not requiring medical and surgical treatment, categorized as the asymptomatic PDA (aPDA) group.

This distinction was made to optimally test both clinically and the machine learning RF model's ability to differentiate hemodynamically significant PDA from aPDA. This grouping is a critical step for evaluating the predictive power of the Random Forest model within the JASP application in distinguishing hemodynamically significant hPDA from non-hemodynamic aPDA.

### 2.3 Data selection

**Figure 1:**
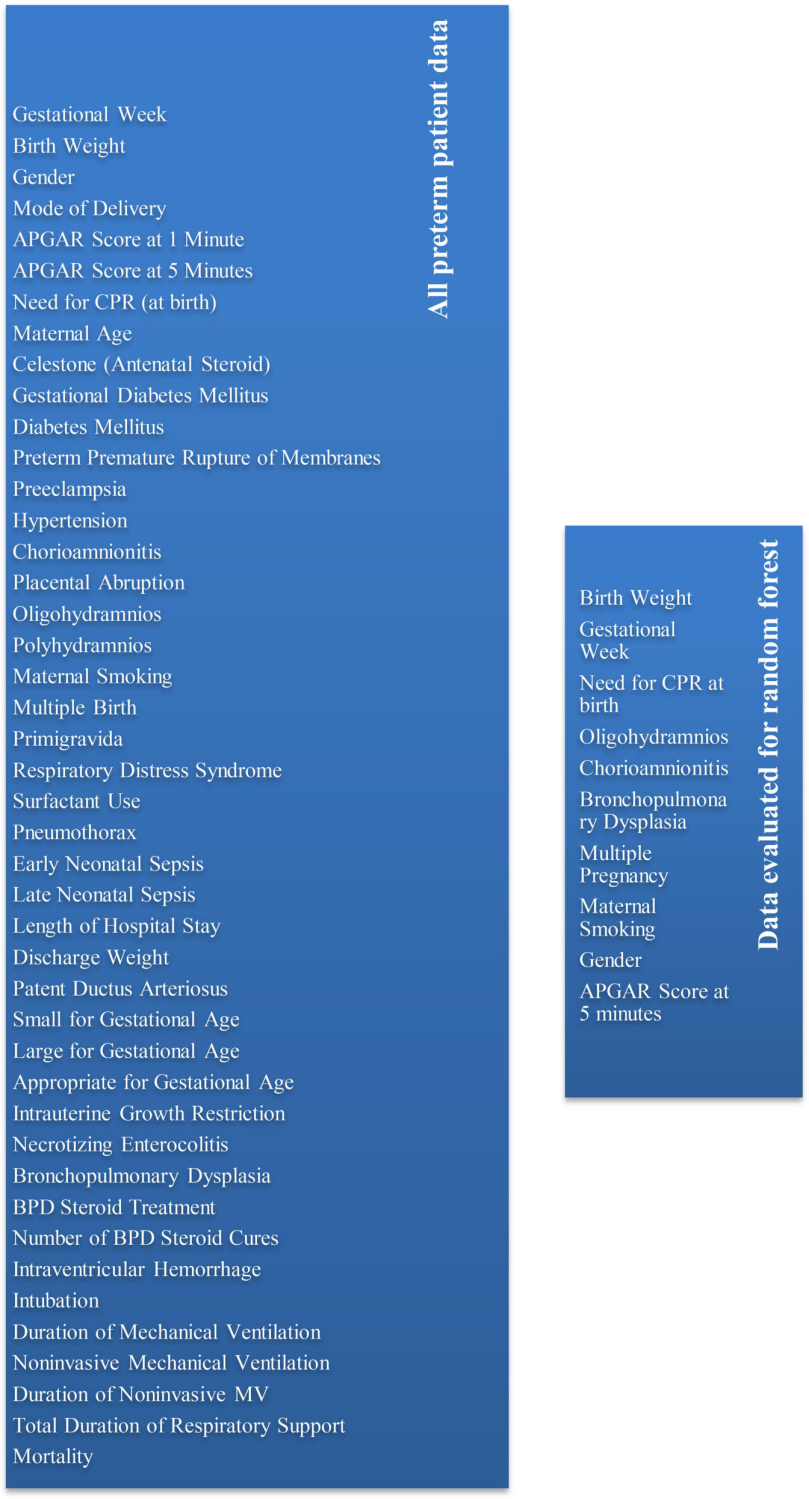
Data selection process.

To rigorously identify the most impactful predictors and optimize the model's performance, the authors subjected the data illustrated in Figure 1 to comprehensive analysis within the Random Forest model, systematically exploring various manual data combinations via the JASP program. From the perspective of the hPDA prediction model, we prioritized prenatal and natal values from these parameters. The variables found to most effectively predict hemodynamically significant PDA(hPDA), necessitating medical or surgical ligation, and distinguishing it from the hemodynamically insignificant aPDA group, included: gender, low birth weight, reduced gestational age, requirement for CPR at birth, Apgar score at five minutes, presence of oligohydramnios, chorioamnionitis, multiple gestations, maternal smoking habits, and the manifestation of bronchopulmonary dysplasia (BPD).

### 2.4 Statistical analysis and machine learning

Statistical analyses and machine learning methodologies were employed to assess the data using the open-source JASP 0.95.2 software (17). Continuous variables were reported as mean ± standard deviation for parametric distributions and as medians for non-parametric distributions; categorical variables were presented as counts and percentages. The Kolmogorov–Smirnov test was performed to ascertain the normality of data distribution. Bivariate relationships between variables were assessed using a simple correlation test. Differences among categorical variables were investigated through chi-square analysis. For quantitative comparisons, Student's t-test and ANOVA were utilized for normally distributed parameters, while the Mann Whitney U and Kruskal Wallis tests were applied for parameters demonstrating non-normal distribution. A *p*-value of <0.05 was designated as statistically significant for all analyses.

The Random Forest algorithm, located under the classification section of the machine learning tab in the JASP program, will be employed for machine learning. The dataset will be allocated as follows: 65% for the training set, 20% for the validation set, and 15% for the test set. Key metrics to be recorded include Support, Accuracy, Precision, Recall, False Positive Rate, False Discovery Rate, F1 Score, Matthews Correlation Coefficient, Area Under Curve, Negative Predictive Value, True Negative Rate, and False Negative Rate. Furthermore, for the machine learning model, Mean Decrease in Accuracy, Total increase in Node Purity, and Mean dropout loss values of the features will be documented. This study designates the hemodynamically significant hPDA group (comprising patients treated with medical closure and surgical ligation) and the hemodynamically insignificant, spontaneously resolving aPDA group as the target variables.

## 3 Results

### 3.1 Distribution of demographic and clinical variables according to PDA presence

When a total of 657 preterm cases under 37 weeks were examined, hemodynamically PDA was not detected in 487 cases, while it was present in 170 cases.

A comparative analysis of fundamental clinical and demographic variables between infants with hPDA and aPDA revealed notable disparities. Gestational age was significantly reduced in the hPDA cohort, averaging 27(25–28) weeks, in contrast to 29(27–31) weeks in the aPDA cohort (*p* < 0.001). Correspondingly, birth weight was markedly lower in the hPDA group 895(705–1189 gr) vs. an average of 1,120(873–1,357) g in the aPDA group (*p* < 0.001). Assessment of APGAR scores indicated comparable 5th-minute scores across both groups [aPDA: 7(7–8), hPDA: 7(7–8); *p*:0.177], yet 1st-minute scores were marginally depressed in the hPDA group [aPDA: 6(5–7), hPDA: 6(4–7); *p*:0.007]. Clinical course evaluation demonstrated prolonged hospitalization for hPDA cases [aPDA: 49(30–72) days, hPDA: 61(27–92) days; *p*:0.005]. Furthermore, the duration of mechanical ventilation was substantially elevated in the hPDA group [aPDA: 0(0–2) days, hPDA: 2(0–7) days; *p* < 0.001]. (Table 1)

**Table 1:** Statistical results of descriptive variables.

Variable		aPDA (*n*: 487)	hPDA (*n*: 170)	*p* value
Gestational week		29 (27–31)	27 (25–28)	**<0.001**
Birth weight (gr)		1,120 (873–1,357)	895 (705–1,189)	**<0.001**
APGAR Score at 1 min		6 (5–7)	6 (4–7)	**0.007**
APGAR Score at 5 min		7 (7–8)	7 (7–8)	0.177
Length of hospital stay (day)		49 (30–72)	61 (27–92)	**0.005**
Duration of mechanical ventilation (intubation) (day)		0 (0–2)	2 (0–7)	**<0.001**
Gender	Male (*n*: 328)	246	82	0.609
	Female (*n*: 329)	241	88	
CPR at birth	No (*n*:564)	439	125	**<0**.**001**
	Yes (*n*:92)	47	45	
Oligohydramnios	No (*n*: 613)	457	156	0.351
	Yes (*n*:44)	30	14	
Chorioamnionitis	No (*n*: 642)	478	164	0.206
	Yes (*n*: 15)	9	6	
Multiple Birth	No (*n*: 441)	337	104	0.051
	Yes (*n*: 215)	149	66	
Maternal Smoking	No (*n*:598)	451	147	**0**.**016**
		Yes (*n*: 59)	36	23
Bronchopulmonary Dysplasia	No (*n*: 370)	306	64	**<0**.**001**
	Yes (*n*: 287)	181	106	

Values are expressed as median with the interquartile range (first quartile – third quartile) in parentheses. CPR, cardiopulmonary resuscitation.

Statistically significant *p*-values are indicated in bold.

Regarding the gender distribution in Table 1, the aPDA group comprised 246 males and 241 females, while the hPDA group included 82 males and 88 females, with no significant difference observed between the two groups in terms of gender (*p* = 0.609). There were 92 cases requiring CPR at birth, and 45 of these had hPDA, which yielded a statistically significant result in the hemodynamically significant hPDA group (*p* < 0.001). Oligohydramnios was present in 30 cases in the aPDA group and 14 cases in the hPDA group (*p*:0.351); conversely, cases without oligohydramnios were dominant in both groups. Similarly, the presence of chorioamnionitis was detected in a limited number of cases in both groups, and mostly this condition was not observed (*p*:0.206). No significant difference was found when examining the multiple pregnancy variable (*p*:0.051). Concerning maternal smoking, a history of smoking was found in 36 cases in the aPDA group and 23 cases in the hPDA group, which was statistically significant (*p*:0.016). When evaluating the distribution of bronchopulmonary dysplasia, among a total of 287 patients diagnosed with BPD, 106 had hPDA, and this also resulted in a higher prevalence of BPD in all patients with hPDA, which was statistically significant (*p*:<0.001; Table 1).

### 3.2 Machine learning results

**Figure 2:**
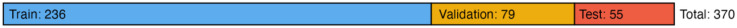
Random forest data split numbers.

In the analysis conducted using the Random Forest classification model, generated by running the JASP program on the dataset comprising 657 registered patients, the JASP program automatically evaluated a total of 370 cases. The data were automatically split within the JASP program into a training set (63.7%), a validation set (21%), and a test set (14.8%). The model's accuracy rate was found to be 91.7% on the test set (Table 2, Figure 2).

**Table 2:** Model summary: random forest classification.

Trees	Features per split	*n* (Train)	*n* (Test)	Test accuracy	OOB accuracy
50	3	236	55	0.917	0.250

Upon examining the confusion matrix, 42 of the 45 cases aPDA were correctly classified, while 3 cases were incorrectly classified as hPDA. All 10 cases with existing hPDA were correctly predicted. This result indicates that the model has high sensitivity, especially in distinguishing hPDA cases (Table 3).

**Table 3:** Confusion matrix.

		Predicted	
		aPDA	hPDA
Observed	aPDA	42	3
	hPDA	0	10

Number of tested cases (total case *n*: 55).

In the model's performance evaluation, accuracy was found to be 91.7%. The high Area Under the ROC curve (AUC = 0.950) indicates significant discriminative power between the two classes. The Recall value was found to be 100% for the hPDA group (no false negatives) and 93.3% for the aPDA group. Precision values were 100% for hPDA negative and 76.9% for hPDA positive, suggesting a limited number of false positives in the positive class. F1 scores were 0.947 (negative), 0.800 (positive), and an average of 0.923, respectively, revealing that the model exhibits a balanced and strong performance in terms of both sensitivity and precision, especially with a very low risk of missing clinically critical positive cases. These results indicate that the model is particularly strong in detecting hPDA positive cases, but its precision value is somewhat limited due to the relatively low number of positive class instances. A negative predictive value of 66.7% suggests that the model's assessment of aPDA group should be evaluated more carefully (Table 4).

**Table 4:** Random Forest performance results.

Model performance metrics	aPDA	hPDA	Average/Total
Support	42	13	55
Accuracy	0.917	0.917	0.917
Precision (Positive predictive value)	1.000	0.769	0.944
Recall (True positive rate)	0.933	1.000	0.923
False positive rate	0.000	0.100	0.050
False discovery rate	0.000	0.231	0.115
F1 score	0.947	0.800	0.923
Matthews correlation coefficient	0.775	0.775	0.775
Area under curve (AUC)	0.950	0.950	0.950
Negative predictive value	0.667	1.000	0.833
True negative rate	1.000	0.900	0.950
False negative rate	0.100	0.000	0.050
False omission rate	0.333	0.000	0.167
Threat score	9.000	1.000	5.000
Statistical parity	0.750	0.250	1.000

All metrics are calculated for every class against all other classes.

When feature importance metrics were analyzed, birth weight and gestational week emerged as the most influential factors within the model. These variables demonstrated the highest contributions to both node purity increase and mean decrease in accuracy criteria. Furthermore, while the impact of birth resuscitation need, oligohydramnios, chorioamnionitis, bronchopulmonary dysplasia, multiple pregnancy, maternal smoking, gender, and the 5th-minute APGAR score was comparatively lower, it is notable that the mean dropout loss value for the APGAR score was relatively high. In this context, variables such as oligohydramnios, chorioamnionitis, BPD, multiple pregnancy, gender, and 5th-minute Apgar score, which had zero Mean decrease in accuracy and Total increase in Node Puirty values, exhibited high Mean dropout loss scores. Although these data were less influential in the Random Forest tree, they were retained in the final model due to their elevated Mean dropout loss values. These findings underscore that birth weight and gestational week are the most robust predictors for assessing PDA risk in neonates, concurrently indicating that other factors provide modest yet significant contributions to the model's overall predictive capability (Table 5).

**Table 5:** Variables used in Random Forest.

Feature importance metrics	Mean decrease in accuracy	Total increase in node purity	Mean dropout loss
Birth weight	−0.004	0.065	0.433
Gestational week	0.011	0.051	0.418
Need for CPR at birth	−0.001	0.007	0.264
Oligohydramnios	4.167 × 10^−4^	0.000	0.245
Chorioamnionitis	0.000	0.000	0.244
Bronchopulmonary Dysplasia	0.000	0.000	0.245
Multiple pregnancy	0.000	0.000	0.244
Maternal smoking	−0.003	−0.002	0.241
Gender	−0.001	−0.003	0.269
APGAR score at 5 min	−0.005	−0.010	0.291

Mean dropout loss [defined as 1 - area under curve (AUC)] is based on 50 permutations.

## 4 Discussion

Our study utilized Random Forest assessment within the machine learning framework. Prior to Random Forest modeling, an evaluation of these data using classical statistical methods indicated that *p*-values were not consistently significant across all variables. Specifically, while gestational week, birth weight, maternal smoking, CPR requirement, 1st-minute APGAR score, and BPD demonstrated statistical significance, factors such as gender, 5th-minute APGAR score, oligohydramnios, chorioamnionitis, and multiple pregnancy were deemed insignificant (Table 1). The Random Forest model effectively identified a range of significant variables, including those like gender, 5th-minute APGAR score, oligohydramnios, chorioamnionitis, and multiple pregnancy, which traditional statistical analyses had previously dismissed as insignificant. This striking divergence underscores the Random Forest model's superior capacity to discern intricate, complex relationships that are often overlooked by conventional approaches, thereby establishing a critical and previously unappreciated role for these factors in hPDA prediction. Our research illustrates how machine learning can convert statistically non-significant data into valuable insights. This underscores the capacity of machine learning models to uncover latent patterns and multi-variable interactions within biomedical datasets, particularly where conventional statistical analyses prove inadequate (8). Consequently, this phenomenon in machine learning warrants further in-depth investigation through new studies, with a call for more comprehensive analysis of data across medical and other domains. These findings robustly affirm that the Random Forest model can offer predictive capabilities extending beyond classical statistical analyses, making substantial contributions to clinical decision-making, particularly in pediatric populations and intricate pathophysiological contexts (18).

In the evaluation of outcomes derived from the Random Forest model, the F1 score and Matthews Correlation Coefficient scores are highlighted as prominent metrics. Research in the existing literature underscores the crucial role of F1 score and MCC values in assessing model efficacy (19, 20). F1 score and MCC values approaching 1 indicate a higher predictive probability of the model. Beyond solely considering MCC and F1 scores, the inclusion of AUC results in the evaluation of machine learning models contributes to improved accuracy. The AUC, defined as the area beneath the Receiver Operating Characteristic curve, encapsulates a model's performance across its full range of classification thresholds and demonstrates greater resilience to class imbalances (21). The text points to existing literature that underscores the criticality of these metrics, citing an example from a heart failure classification study where a Random Forest method achieved high accuracy with an AUC of 0.97 and an MCC of 0.83 (22). Regarding the current study's findings, the Random Forest model demonstrated strong performance with an F1 score of 92.3%, an MCC of 77.5%, and an AUC of 95%, indicating high sensitivity and precision. The text also notes that similar studies in the literature show high success rates for Random Forest models in complex medical diagnostic problems (23). A comparison is drawn to another Random Forest model for PDA risk prediction in preterm infants, which reported an acceptable performance with 76.3% accuracy and 90% specificity (8). The author suggests that their Random Forest model exhibits superior performance in both sensitivity and specificity compared to similar studies, implying it could be a more reliable tool in clinical practice.

In our study, among the data used in the Random Forest model, gestational week, birth weight, and the need for resuscitation at birth were identified as significant predictor variables (Table 5). These findings play a critical role in developing strategies for early diagnosis and treatment, and similarly, the literature confirms that low birth weight and low gestational age are important predictors in identifying neonates at risk for patent ductus arteriosus (8). Additionally, the study by Park et al. yielded results similar to ours. This study showed that infants in the symptomatic PDA group had statistically significantly lower gestational age, lower birth weight, and lower 1st-minute and 5th-minute Apgar scores (1). Indeed, previous different studies have also demonstrated that these parameters are strong predictors for the risk of hPDA development, especially in very low birth weight infants (24). Furthermore, some studies have indicated that each one-week increase in gestational age reduces the probability of PDA, and low birth weight is associated with a high risk of PDA (8). These results are further strengthened by artificial intelligence, highlighting the potential of machine learning models like Random Forest for PDA risk assessment in premature infants. As a result of these studies, more stable artificial intelligence models can be developed to monitor patients more precisely, and potential complications can be predicted early for timely intervention. Specifically, by preventing hemodynamically insignificant PDA with potential for early spontaneous closure from undergoing unnecessary invasive treatment, significant contributions can be made to clinical decision-making processes. Moreover, AI-supported models will enable more personalized and effective treatment plans by creating customized risk profiles based on each patient's individual characteristics (25).

The assessment of feature importance within Random Forest modeling is significantly informed by metrics such as Mean Decrease in Accuracy, Total Increase in Node Purity, and Mean Dropout Loss, which offer critical insights into the model's operational mechanisms (26, 27). These metrics are instrumental in discerning the features that maximally contribute to the model's predictive efficacy, thus articulating the differential relevance of various variables (26). Our investigation identified gestational week, birth weight, and the requirement for resuscitation at birth as paramount determinants within the model, exhibiting substantial contributions to both the augmentation of node purity and the mitigation of Mean Decrease in Accuracy. This observation robustly substantiates the pivotal role of these three core parameters in prognosticating the risk of hemodynamically significant patent ductus arteriosus in preterm neonates. Conversely, the comparatively elevated Mean Dropout Loss for the APGAR score, despite a lesser contribution when juxtaposed with other importance metrics, signifies its non-negligible influence on the model's aggregate performance. Furthermore, variables including oligohydramnios, chorioamnionitis, bronchopulmonary dysplasia, and multiple pregnancy, while displaying a Mean Decrease in Accuracy of 0 and possibly limited effectiveness within the RF model, remain integral foundational data. This caliber of quantitative and qualitative feature importance analysis diminishes the inherent opacity of the model, enabling clinicians to comprehend the most impactful predictive factors and thereby augmenting the model's trustworthiness in clinical decision-making. Consequently, the model's demonstrated high performance, coupled with its robust interpretability and transparency derived from detailed feature importance analysis, not only reinforces its reliability but also significantly enhances its trustworthiness and potential for practical application within the critical medical domain.

Future studies of this nature require support through multi-center, large-cohort studies to facilitate their integration into prospective clinical practice and enhance their generalizability (4). This study, which can be considered a precursor to prospective research, clearly demonstrates the potential of machine learning models to contribute to diagnostic and risk assessment processes in pediatric cardiology (28–31). Future investigations are imperative for further advancing the predictive capacity of this model by integrating richer datasets, such as additional biomarkers or genomic data, which will substantially enhance its prognostic accuracy. These integrations will enable the development of more personalized and accurate approaches for the detection and management of hemodynamically significant PDA in preterm infants (30). Consequently, continued investigation into comparative analyses of diverse algorithmic frameworks and the interpretability of predictive models is imperative. Such efforts are critical for expanding the clinical applicability of machine learning in pediatric cardiology and optimizing its therapeutic advantages (8).

## 5 Conclusion

This study's findings underscore the Random Forest model's efficacy in evaluating the risk of hemodynamically significant patent ductus arteriosus in premature infants, thereby presenting substantial contributions to clinical utility. The conducted analyses identified factors such as low birth weight, reduced gestational age, and the necessity for CPR at birth as pivotal predictors for PDA development risk. These insights are paramount for the timely identification of neonates at risk and the formulation of suitable therapeutic strategies, allowing for early risk assessment of PDA in neonatal intensive care units, which in turn facilitates the creation of targeted interventions and enhances patient prognosis. Nevertheless, the full integration of these models into prospective clinical applications mandates further validation and reinforcement through extensive, multi-center prospective investigations. As the domains of artificial intelligence and machine learning progressively evolve, the incorporation of such prognostic models into clinical decision support systems is poised to significantly amplify the role of AI in both research endeavors and clinical applications.

## Data Availability

The original contributions presented in the study are included in the article/Supplementary Material, further inquiries can be directed to the corresponding author.
